# The impact of malignant nipple discharge cytology (NDc) in surgical management of breast cancer patients

**DOI:** 10.1371/journal.pone.0182073

**Published:** 2017-08-14

**Authors:** Isabella Castellano, Jasna Metovic, Davide Balmativola, Laura Annaratone, Nelson Rangel, Elena Vissio, Riccardo Arisio, Luigia Macrì, Carla Pecchioni, Ivana Sarotto, Francesca Montarolo, Francesca Muscarà, Caterina Marchiò, Paola Cassoni, Janina Kulka, Anna Sapino

**Affiliations:** 1 Department of Medical Sciences, University of Turin, Turin, Italy; 2 Fondazione del Piemonte per l'Oncologia (FPO) - Candiolo Cancer Institute (IRCCs), Candiolo, Italy; 3 Natural and Mathematical Sciences Faculty, University of the Rosario, Bogotá, Colombia; 4 Department of Surgical Pathology, AOU Città della Salute e della Scienza of Turin, Turin, Italy; 5 Royal Marsden Hospital Foundation Trust, Department of Breast Surgery, London, United Kingdom; 6 Department of Pathology II, Semmelweis University, Budapest, Hungary; University of North Carolina at Chapel Hill School of Medicine, UNITED STATES

## Abstract

**Background:**

The role of nipple discharge cytology (NDc) in the surgical management of breast cancer patients is unclear. We aimed: (i) to evaluate the effect of malignant NDc on the surgical approach to the nipple-areola complex, and (ii) to verify the association between malignant NDc and nipple malignancy.

**Methods:**

We retrospectively analyzed a case series of 139 patients with NDc who underwent breast surgery. The clinical and histological findings, types of surgery with emphasis on nipple-areola complex amputation, immunohistochemical phenotypes of the carcinomas and measurements of the tumor-nipple distance were recorded. Additionally, in patients who showed HER2-positive lesions on definitive surgery, we evaluated the HER2 immunocytochemistry of the NDc smears.

**Results:**

Thirty-two malignant and 107 benign/borderline NDc diagnoses were identified. All 32 malignant-NDc cases were histologically confirmed as malignant. Thirty borderline/benign-NDc cases were histologically diagnosed as malignant (sensitivity 58%). The majority of the patients with malignant NDc were treated with nipple-areola complex amputations in both the mastectomy and conservative surgery groups (P<0.001, χ^2^51.77). Nipple involvement was strongly associated with HER2-positive ductal carcinoma *in-situ* (P<0.001, χ^2^11.98). HER2 immunocytochemistry on the NDc revealed a 100% correlation with the immunocytochemistry performed on the surgical tissues.

**Conclusions:**

Malignant NDc influenced surgical management. The association of malignant NDc with nipple involvement is highly related to ductal carcinoma in-situ with HER2 overexpression. In case of HER2 positive NDc, nipple-areola complex involvement is more likely than in HER2 negative cases.

## Introduction

Nipple discharge (ND) accounts for approximately 5% of breast-related symptoms [1] and is the third most common reason women seek medical attention, following breast lumps and breast pain [2]. Hormonal diseases, such as hypo and hyperthyroidism and prolactinoma [1, 2], generally cause bilateral ND. In contrast, the majority of unilateral ND cases result from a breast disease, such as intraductal papilloma, duct ectasia or plasma cell mastitis. In addition, approximately 7% to 15% [2] of unilateral NDs are caused by malignant lesions, primarily ductal carcinoma in-situ (DCIS) [2–4] with micropapillary features in which the cells detach in the ducts and flow into the nipple [5]. ND has been classified based on its appearance as milky, yellow, watery, pink or bloody [6, 7]. A meta-analysis demonstrated that bloody ND is a predictor of breast cancer (BC) risk [7]. The role of the cytological results of ND samples in the planning of surgical approaches for patients remains unsettled. Some authors consider it together with clinical and radiological assessment as a diagnostic evaluation of the risk of underlying carcinoma [8], however others recommend to exclude ND cytology (NDc) from the diagnostic algorithms of breast diseases to avoid confusion in patient surgical management [9], whereas others include it but do not give recommendations in case of malignant NDc results [10]. As a matter of fact, the cytological diagnosis of ND may be difficult because the quality of smears may be poor and the number of diagnostic cells may be low, leading to low sensitivity of the procedure [11–13]. On the other hand, the diagnosis of malignant NDc is highly specific (97 to 100%) [12–14]. Few studies are available on the use of NDc results to address surgical techniques on the nipple-areolar complex (NAC). Discouraging results have been published by Cabioglu N et al. [15] and Chang and Cheung [16] on the use of ND as a marker of NAC involvement, however in both studies few cases were examined by NDc and the majority were within the benign category [16]. Nevertheless, new surgical techniques that improve cosmetic results, such as nipple-sparing breast surgery [17–19], require additional information about the presence of occult tumor cells at the NAC site. For example, Paget’s disease of the nipple may be silent at both, clinical examination and radiological imaging.

The Italian Guidelines for nipple sparing mastectomy [20] consider the distance between the lesion and the NAC as the most important parameter for NAC preservation or amputation. In addition, one of the absolute contraindications for nipple preservation is malignant NDc. Thus, in clinical practice it remains uncertain whether this data may be relevant for surgical approach.

Considering this complex background, the aim of this study was twofold: (i) to determine the correlation between malignant NDc and surgical approach to NAC in our retrospective case series; and (ii) to verify the association between malignant NDc and presence of malignancy in less than 2 cm from NAC.

## Materials and methods

We retrospectively selected a series of 139 patients with NDc who underwent surgical breast resection at the Breast Unit of Città della Salute e della Scienza, Molinette Hospital and St. Anna Hospital, Turin, Italy from January 2010 to December 2015.

In our Breast Unit, NDc are smeared on two or more slides, which are then air dried and stained with Giemsa. For the study purposes, the ND smears were re-examined by two pathologists who were blinded to the original cytological diagnosis. The samples were classified in three categories: malignant (cancer cells in the smear), borderline (atypical clusters of epithelial cells disposed in papillary structures and/or sporadic epithelial morulae) and benign (presence of histiocytes, amorphous material, but no epithelial cells). Data regarding ND appearance (bloody *vs* not bloody, mono or pluri-ductal involvement, spontaneous or induced) were collected together with imaging data (i.e. US, mammography and quadrant involvement) and type of surgery (conservative vs mastectomy) (Table 1).

**Table 1:** Clinical and imaging data at presentation of patients with nipple discharge cytology (NDc).

NDc	Benign 62 (45%)	Borderline 45 (32%)	Malignant 32 (23%)	Total 139	*P* value
**Age** (years)					
<40	6 (60%)	1 (10%)	3 (30%)	10	0.01
40–50	16 (53%)	6 (20%)	8 (27%)	30	
51–70	30 (45,5%)	28 (42,5%)	8 (12%)	66	
**>**70	10 (30%)	10 (30%)	13 (40%)	33	
**Color**					
Bloody	33 (37%)	32 (35%)	25 (28%)	90	0.06
Serous	25 (64%)	9 (23%)	5 (13%)	39	
Others *(Serous*, *Milky or Yellow)*	4 (40%)	4 (40%)	2(20%)	10	
**Ductal Involvement**					
Mono-ductal	54 (44%)	41 (33%)	28 (23%)	123	0.79
Pluri-ductal	8 (50%)	4 (25%)	4 (25%)	16	
**Presentation**					
Spontaneous	54 (45%)	41 (34%)	26 (21%)	121	0.44
Induced	8 (44,5%)	4 (22%)	6 (33,5%)	18	
**Mammography**					
Positive (R4/R5)	18 (36%)	5 (10%)	27 (54%)	50	<0.001
Doubt (R3)	22 (61%)	13 (36%)	1 (3%)	36	
Negative (R1/R2)	22 (41%)	27 (51%)	4 (8%)*	53	
**Ultrasound**					
Positive (U4/U5)	17 (34%)	5 (10%)	28 (56%)	50	<0.001
Doubt (U3)	24 (58%)	16 (39%)	1 (3%)	41	
Negative (U1/U2)	21 (44%)	24 (50%)	3 (6%)	48	
**Quadrant involvement**					
Central quadrant	38 (41%)	39 (43%)	15 (16%)	92	<0.001
Others	24 (51%)	6 (13%)	17 (36%)	47	
**Type of surgery**					
Mastectomy	7 (28%)	0	18 (72%)	25	<0.001
Conservative	55 (48.5%)	45 (39.5%)	14 (12%)	114	

*three out four patients were negative for both Mammography and Ultrasound.

The surgical specimens of patients with ND were re-examined and the lesions were classified as benign (papillary/hyperplastic lesions) and malignant (in-situ/invasive cancers). We re-assessed their size and histological type. In case of malignancy, immunophenotype (ER, PR, HER2 and KI67) was obtained from diagnostic report. According to the local protocol, NAC surgical specimen is analyzed on at least two different paraffin embedded blocks. For the study purposes, we evaluated 5 extra hematoxylin-eosin stained sections from each block to better define the distance from the lesion to the tip of the nipple. Taking into account the minimal distance recommended by guidelines for considering nipple-sparing surgical approach [20], we considered involvement of NAC when the lesion was ≤2 cm from the tip of the nipple either in the form of intraductal spreading or of stromal invasion. Ethical approval for this study was obtained from the Comittee for human Biospecimen Utilization (Department of Medical Sciences -ChBU). The project provided a verbal and not written informed consent, obtained at the time of surgery, from the patients due to the retrospective approach of the study, which did not impact on their treatment. The procedure for collecting verbal consent was approved by the Committee for human Biospecimen Utilization (Department of Medical Sciences -ChBU). All the cases were anonymously recorded and data were accessed anonymously.

### Immunohistochemistry of NDc

In the cases of HER2-positive malignant lesions at the definitive surgery, considering that HER2 is the biomarker used to highlight the presence of cancer cells in nipple-areola area [21–25], we evaluated the HER2 status of the corresponding ND smears. Specifically, one slide for each case was de-mounted, and endogenous peroxidase was blocked by incubation with 6% H_2_O_2_. The slides were then incubated with the primary antibody included in the HercepTest^™^ (Dako, Glostrup, Denmark) for 30 minutes at room temperature. After washing, the slides were incubated with Dako EnVision^™^Systems solution. The reaction was developed in a solution containing 3,3’diaminobenzidine (LiquidDAB Substrate Pack, BioGenex. Freemont, Ca). HER2 intensity was scored following ASCO/CAP guidelines [26] and NDc were considered as positive if an intense to moderate HER2 staining was present on the membrane of at least ten morphologically malignant cells.

As controls of the specificity of HER2, immunocytochemical (ICC) reaction, 3 negative and 5 borderline ND smears, which were not correlated with malignancy on NAC histological specimens, were stained following the same procedure.

In addition, two air dried smears stained with Giemsa of HER2-positive BT474 and HER2-negative MCF7 BC cell lines were prepared and one week later were demounted and were processed as above reported.

### Statistical analysis

Statistical analyses were performed using contingency tables (Chi Square test and Fisher Exact test). The statistically significant probability value was set at P<0.05. We calculated at that point the sensitivity, specificity and positive (PPV) and negative (NPV) predictive values of the NDc.

## Results

As shown in Table 1, of the 139 NDc smears, 32 were malignant, 45 were borderline, and 62 were benign. In the majority of patients, ND was bloody, spontaneous and mono-ductal.

Malignant NDc correlated with a high suspect of breast malignancy at ultrasound and/or mammography examination (*P*<0.001). DCIS were detected in 3 patients studied by magnetic resonance with malignant NDc and negative mammography and ultrasound results. Two of these were DCIS with micropapillary features and one was a solid DCIS with intra-ductal papilloma.

The correlation between NDc and histological diagnosis of surgical specimens confirmed that 100% of malignant NDc were related to an in-situ and/or invasive carcinoma. Otherwise, only 28% (30/107) of benign/borderline NDc were related to malignancy. In the remaining 72% (77/107) of cases a papillary or hyperplastic intraductal proliferation was diagnosed (Table 2).

**Table 2:** Correlation between NDc and histological diagnosis of surgical specimens.

	ND Cytology			*P* value
	Total 139	Benign+Borderline 107	Malignant 32	
***Histological diagnosis***				
**Malignant**	**62 (45%)**	**30 (28%)**	**32 (100%)**	< 0.001
DCIS^a^	29	12	17	
DCIS and IC^b^	22	10	12	
IC^b^	11	8	3	
**Non malignant**	**77 (55%)**	**77 (72%)**	**0**	
Benign Lesion	41	41	/	
Papillary Lesion	36	36	/	

^a^DCIS: ductal carcinoma in-situ.

^b^IC: Invasive carcinoma.

The specificity and the PPV of NDc were 100% with a sensitivity of 58% and a NPV of 63%. The malignant lesions were generally larger in the cases with malignant NDc (P <0.001) (Table 3).

**Table 3:** Correlation between NDc and histological size of surgical specimens.

	ND Cytology		P value
Size of lesion	Benign /Borderline 107	Malignant 32	
**<20 mm**	85	12	< 0.001
**20–30 mm**	13	8	
**>30 mm**	2	10	
**Multicentric**	2	2	

### Correlation between malignant NDc and surgical approach to NAC

**Fig 1:**
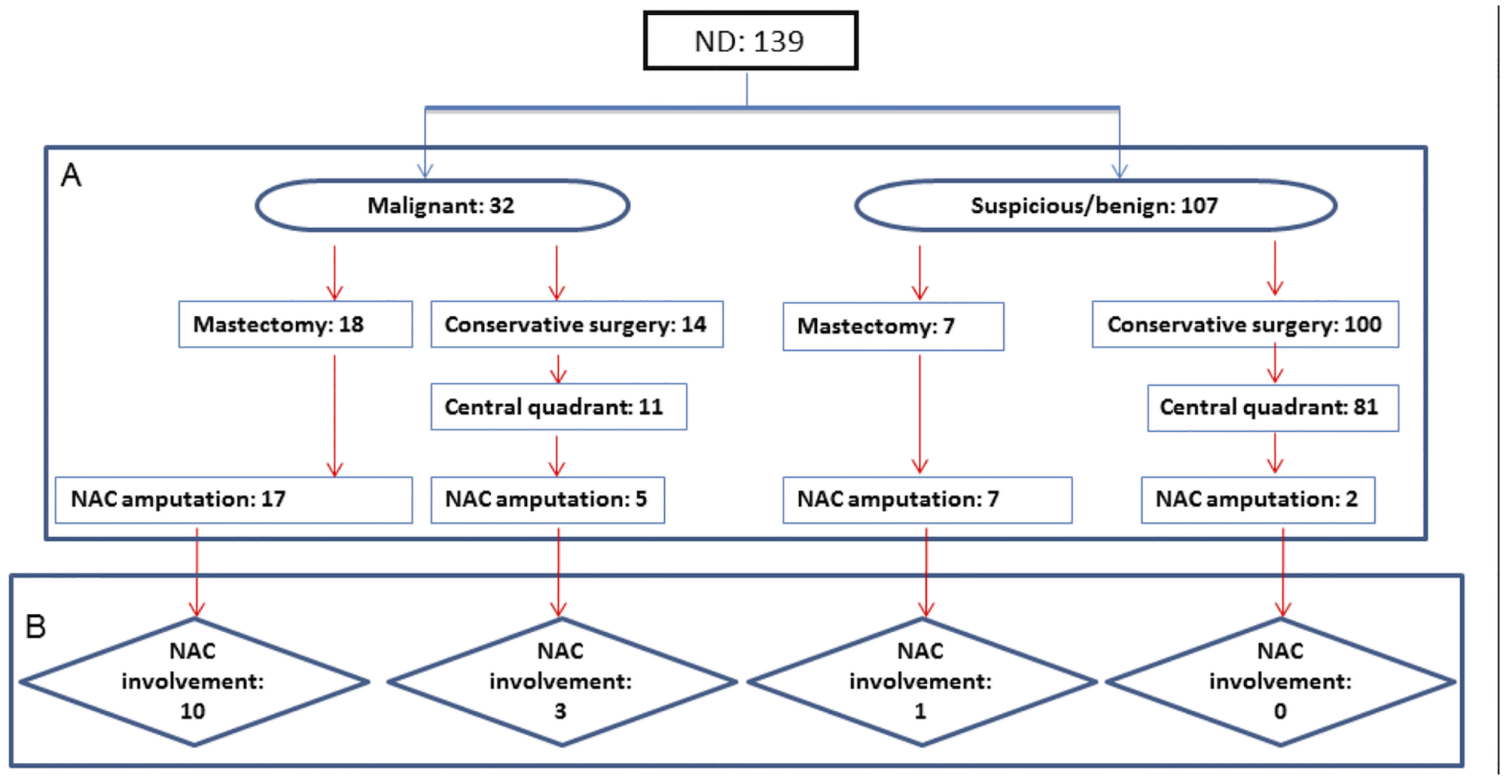
Nipple discharge cytology (NDc). Summary of results, types of surgery and nipple areola complex (NAC) involvement at histology.

Fig 1 provides a summary of the NDc results, surgical approach and NAC status at histology. Of the 32 patients with malignant NDc, in 22 cases NAC amputation was performed, 17 during mastectomy and 5 during conservative surgery with central quadrant excision. On the other hand, in borderline/benign NDc, NAC amputation was performed in all cases with mastectomy and in 2/81 central quadrant excision surgeries (Fig 1A). Approximately, 70% of the patients (22/32) with malignant NDc and 8.4% (9/107) with benign/borderline NDc were treated with NAC amputation (P<0.001).

### Association between malignant NDc and presence of malignancy in less than 2 cm from NAC

To clarify whether a malignant NDc per se might have a role in the selection of patients who require NAC amputation, we analyzed in detail the specimens of the 31 patients (24 mastectomy and 7 conservative surgery) undergoing this surgical procedure (Fig 1A) and correlated it with the NDc results and histological characteristics (Table 4).

**Table 4:** Pathology data of the malignant breast lesions of patients who underwent nipple-areola complex (NAC) amputation.

	NAC involved 14 (45%)	NAC free 17 (55%)	Total 31	*P Value*
**NDc**^a^				0.014
Malignant	13	9	22	
Benign/Borderline	1	8	9	
**Type of lesion**				0.90
DCIS^b^	6	6	12	
DCIS + IC^c^	6	10	16	
Invasive carcinoma	2	1	3	
**Histological Grade of IC**^c^				0.44
1	3	3	6	
2	0	4	4	
3	5	4	9	
**Histological type of DCIS**^d^				0.91
Micropapillary	5	7	12	
Papillary	3	3	6	
Cribriform	1	1	2	
With comedonecrosis	3	5	8	
**Nuclear grade of DCIS**^e^				0.76
Low	0	1	1	
Intermediate	2	3	5	
High	4	2	6	
**HER2 on histological specimens**				<0.001
Positive	9	1*	10	
Negative	5	16	21	
**Estrogen Receptor**				0.76
>1	11	15	26	
0	3	2	5	
**Progesterone Receptor**				0.62
>1	4	7	11	
0	2	1	3	
Not Known	8	9	17	
**Size of cancer at histology**				0.94
<20 mm	5	6	11	
20–30 mm	4	7	11	
>30 mm	5	4	9	
**HER2 on malignant NDc**				<0.001
Positive	9	0	9	
Negative	5	17	22	

^a^NDc: nipple discharge cytology;

^b^DCIS: ductal carcinoma in-situ;

^c^IC: invasive carcinoma;

^d^DCIS both as single lesion or associated at invasive carcinomas;

^e^DCIS as pure lesion;

*HER2 was positive only in the infiltrating carcinoma.

**Fig 2:**
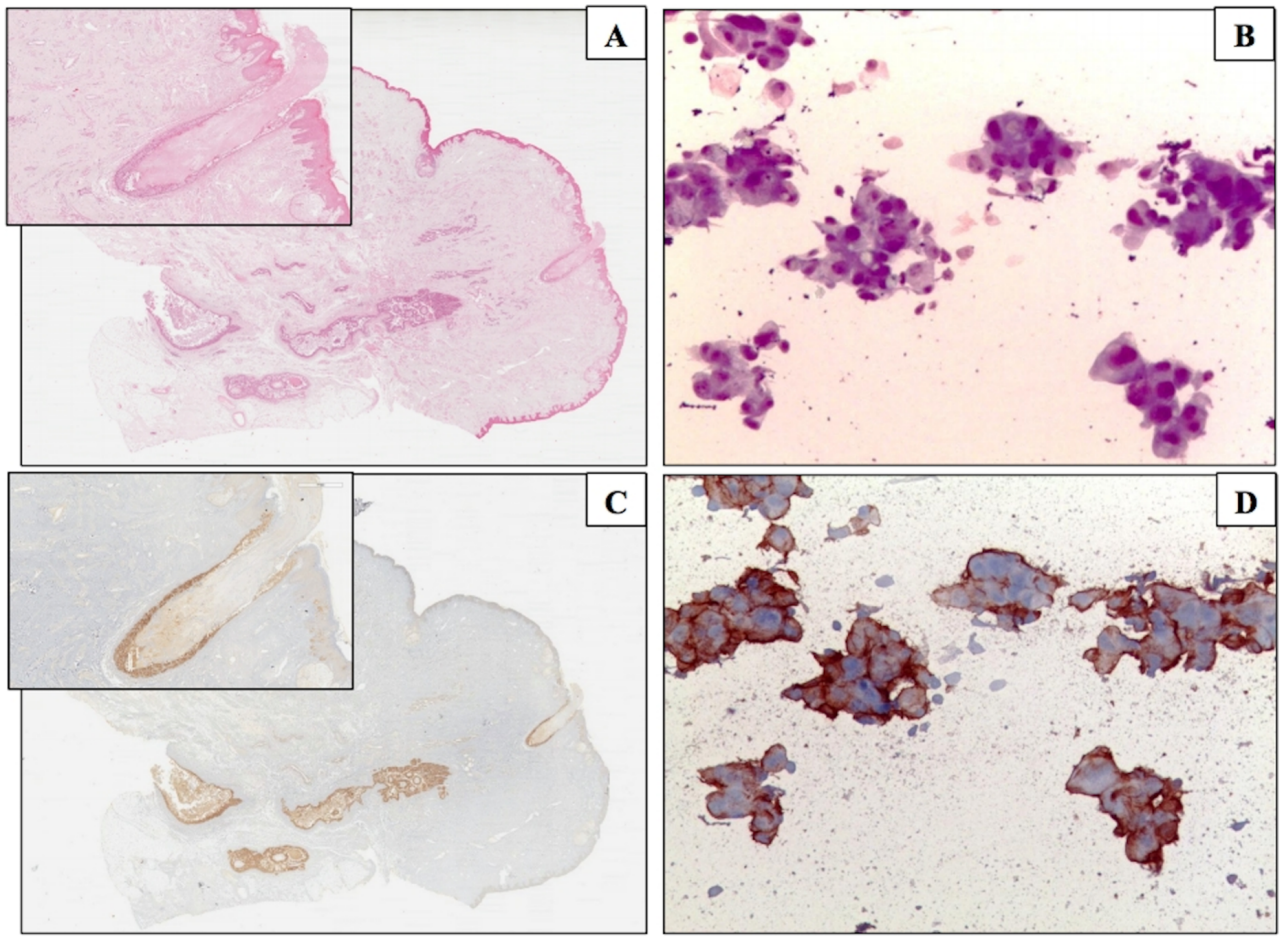
Giemsa staining & ICC. Giemsa staining on ND smear (A) and on the corresponding DCIS (B). HER2 ICC expression of the malignant cells on ND smear (C) and on the corresponding DCIS (D).

Of the 31 patients with NAC amputation, 14 had NAC involved by DCIS and/or invasive cancer (Fig 2A) and 13 of these had a malignant NDc (*P* 0.014) (Fig 2B).

**Fig 3:**
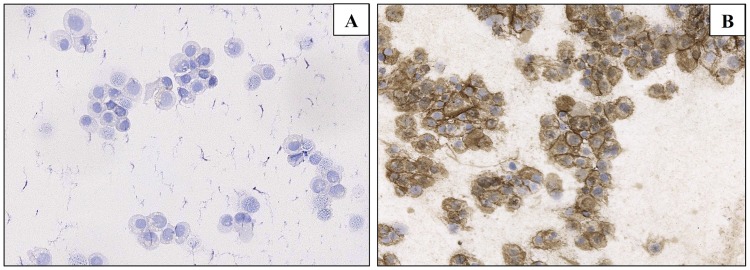
HER2 expression in BC cell lines. Immunohistochemistry for MCF7 (A) and BT474 (B) cells.

HER2 was overexpressed (score 3+) in 9 cases. All of these were DCIS (Fig 2C), growing within lactiferous ducts of the subareolar region and/or the nipple and showing malignant NDc. The immunostaining of the corresponding NDc smears demonstrated that the malignant cells overexpressed HER2 as well (Table 4) (Fig 2D). In the subset of 17 cases without NAC involvement, only 1 (6%) invasive cancer exhibited HER2 overexpression (*P*<0.001); however, both the associated DCIS and the corresponding NDc were HER2-negative. HER2 membrane expression was not detected on epithelial cells of negative and suspicious NDc smears. The immunostaining performed as control confirmed MCF-7 cells as negative for HER2 overexpression (Fig 3A), while BT474 cells showed a positive membrane staining, but some granules were immunostained within the cell cytoplasm as well (Fig 3B).

## Discussion

Some studies suggest that NDc should not be considered in the management of patients [10, 9] because, although its high specificity, it shows a low sensitivity [13, 14]. In the present study, we confirmed the low sensitivity of non-malignant NDc (28% had an underlying malignant lesion). On the other hand, we confirmed the high specificity of malignant NDc, that was demonstrated by the presence of an in-situ and/or an invasive carcinoma at definitive surgery in all patients. We than wanted to verify the association between malignant NDc and the real presence of cancer in the nipple area. We found that 13/14 cases with NAC involvement presented malignant NDc.

Moreover, in agreement with others [27], we showed that malignant NDc was frequently correlated with large breast cancers primarily located in the central quadrant.

In clinical practice, there is no consensus regarding the surgical approach to patients with malignant cells on NDc. However, the data we obtained from a retrospective cohort of patients revealed that the majority of cases with malignant NDc underwent NAC amputation irrespective of the type of surgery performed (i.e., mastectomy or conservative surgery). These data suggest that malignant NDc diagnosis may influence surgeons in their clinical practice.

We are aware that our case series is small to draw definitive conclusions, however, our findings are in line with previous works, showing that in-situ carcinomas are the primary cause of NAC involvement and that these DCIS frequently overexpress HER2 [28–30, 25]. Bauer *et al*. [27] showed that when ND is the result of DCIS, in 63% of the cases the central location and the intraductal spreading of cells may preclude breast conserving surgery. In a previous study on micropapillary DCIS, we have demonstrated that this growth pattern may represent a risk factor for local recurrence after breast-conserving surgery and that patient may suffer of spontaneous ND [5], positive at cytology examination. In the present study, we observed that micropapillary and papillary DCIS histotype are frequently related to ND, although this association was not specific of NAC involvement (Table 4).

The rate of occult NAC involvement reported in patients with invasive BC, is highly variable (from 0% to 27%) [31, 32, 18, 33] and mammographic distance between tumor and nipple, axial tumor-NAC distance at magnetic resonance imaging [31, 32], tumor size, pathologic staging [34, 35] and HER2 amplification [33] have been proposed to predict NAC status. The Italian Guidelines for nipple sparing mastectomy [20] consider the distance between the lesion and the NAC evaluated using imaging analysis as the most important parameter for NAC preservation or amputation. A study on imprint cytological assessment of the subareolar tissue showed that it might not be sufficient as an exclusive method for the intraoperative assessment of the NAC, having the sensitivity of 50% and the specificity of 87.58% [36]. The intraoperative assessment of NAC by frozen sections is more sensitive (92%), but to obtain reliable results it is needed to use specific diagnostic protocols [37].

In addition, we showed for the first time the feasibility of the HER2 immunostaining in air dried ND pre-stained smears, and observed a very high concordance with the results obtained on the immunostaining performed on histological sections of the corresponding surgical sample. This suggests the possibility to implement this method for studying malignant NDc and to use the results as an additional biological parameter for guiding NAC surgery.

In conclusion, we observed that malignant NDc influenced surgical decision in our case series and confirmed that malignant NDc is highly specific for the presence of breast cancer, mainly DCIS. Finally, we showed that if NDc is HER2 positive, NAC involvement is more likely than in HER2 negative cases.
